# Recapitulating phylogenies using *k*-mers: from trees to networks

**DOI:** 10.12688/f1000research.10225.2

**Published:** 2016-12-23

**Authors:** Guillaume Bernard, Mark A. Ragan, Cheong Xin Chan

**Affiliations:** 1 Institute for Molecular Bioscience, The University of Queensland, Brisbane, Australia

**Keywords:** phylogenies, phylogenetic trees, phylogenetic networks, k-mers

## Abstract

Ernst Haeckel based his landmark Tree of Life on the supposed ontogenic recapitulation of phylogeny, i.e. that successive embryonic stages during the development of an organism re-trace the morphological forms of its ancestors over the course of evolution. Much of this idea has since been discredited. Today, phylogenies are often based on families of molecular sequences. The standard approach starts with a multiple sequence alignment, in which the sequences are arranged relative to each other in a way that maximises a measure of similarity position-by-position along their entire length. A tree (or sometimes a network) is then inferred. Rigorous multiple sequence alignment is computationally demanding, and evolutionary processes that shape the genomes of many microbes (bacteria, archaea and some morphologically simple eukaryotes) can add further complications. In particular, recombination, genome rearrangement and lateral genetic transfer undermine the assumptions that underlie multiple sequence alignment, and imply that a tree-like structure may be too simplistic. Here, using genome sequences of 143 bacterial and archaeal genomes, we construct a network of phylogenetic relatedness based on the number of shared *k*-mers (subsequences at fixed length *k*). Our findings suggest that the network captures not only key aspects of microbial genome evolution as inferred from a tree, but also features that are not treelike. The method is highly scalable, allowing for investigation of genome evolution across a large number of genomes. Instead of using specific regions or sequences from genome sequences, or indeed Haeckel’s idea of ontogeny, we argue that genome phylogenies can be inferred using *k*-mers from whole-genome sequences. Representing these networks dynamically allows biological questions of interest to be formulated and addressed quickly and in a visually intuitive manner.

## Introduction

Ernst Haeckel coined the term *Phylogenie* to describe the series of morphological stages in the evolutionary history of an organism or group of organisms ^1^. In his Tree of Life published 150 years ago ^2^, Haeckel postulated that living organisms trace their evolutionary origin(s) along three distinct lineages (Plantae, Protista and Animalia) to a “common Moneran root of autogonous organisms”. In some (but not all) later works (e.g. in 1868 ^3^) he allowed that different Monera may have arisen independently by spontaneous generation. Either way, these views accord with the Larmackian notion of a built-in direction of evolution from morphologically simple “lower” organisms to more-complex “higher” forms ^4^.

Haeckel through his “Biogenetic Law” advocated that “ontogeny recapitulates phylogeny” ^2^: that the embryonic series of an organism is a record of its evolutionary history. Under this view, morphologies observed at different developmental stages of an organism resemble and represent the successive stages (including adult stages) of its ancestors over the course of evolution. Of course, he worked before the advent of genetics and the modern synthesis, and before it was appreciated that information on hereditary is carried by DNA and can be recovered by sequencing and statistical analysis. He could not have foreseen that these DNA sequences code for other biomolecules and control life processes, including his beloved developmental series and organismal phenotype, through vastly complex molecular webs of interactions. Nor could Haeckel have envisaged the scale of phylogenetic analysis that can be carried out today using these DNA sequences across multiple genomes, made possible by the advent of high-throughput sequencing and computing technologies.

Fast-forwarding 150 years, phylogenetic inference based on comparative analysis of biological sequences is now a common practice. The similarity among sequences is commonly interpreted as evidence of homology ^5, 6^, i.e. that they share a common ancestry. From the earliest days of molecular phylogenetics, multiple sequences have been aligned ^7, 8^ to display this homology position-by-position along the length of the sequences. That is, the residues are arranged relative to each other such that the best available hypothesis of homology is achieved at every position (column) of the alignment. By default, it is assumed that the best alignment can be achieved simply by displaying the sequences in the same direction, and inserting gaps where needed (to represent insertions and deletions). This assumption is largely valid when working with highly conserved orthologs of any source, and with exons or proteins of morphologically complex eukaryotes. However, microbial genomes are often affected by recombination and rearrangement ^9^, undermining the assumption of homology along adjacent positions, while lateral genetic transfer would not be represented by a common treelike process ^10– 13^. As Haeckel observed when he drew his Tree ^2^, biological evolution can be anything but straightforward, and these complications have become ever more-complicated ^14, 15^.

Alternative approaches for inferring and representing phylogenies are available. An attractive strategy that addresses the issue of full-length alignability is to compute relatedness among a set of sequences based on the number or extent of *k*-mers (short sub-sequences of a fixed length *k*) that they share. Such approaches avoid multiple sequence alignment, and for this reason are termed *alignment-free*. As opposed to heuristics in multiple sequence alignment, these methods provide exact solutions. Various modifications are available, e.g. the use of degenerate *k*-mers, scoring match lengths rather than *k*-mer composition, and grammar-based techniques; see recent reviews ^16, 17^ for more detail. Methods for inferring lateral genetic transfer have also been developed ^18, 19^. Importantly, evolutionary relationships can also be depicted as a network, with taxa and relationships represented respectively as nodes and edges ^20– 24^, rather than as a strictly bifurcating tree. Using simulated and empirical sequence data, we recently demonstrated that alignment-free approaches can yield phylogenetic trees that are biologically meaningful ^25– 27^. We find that these approaches are more robust to genome rearrangement and lateral genetic transfer, and are highly scalable ^25, 26^, a much-desired feature given the current deluge of sequence data facing the research community ^28^. Here we extend the alignment-free phylogenetic approaches on 143 bacterial and archaeal genomes to generate a network of phylogenetic relatedness, and assess biological implications of this network relative to the phylogenetic tree. The phylogenetic relationships among these genomes have been carefully studied using the standard approach based on multiple sequence alignment ^10^ and an alignment-free approach ^25^; this dataset thus provides a good reference for comparison.

## Methods

Using 143 complete genomes of Bacteria and Archaea ^25^, we inferred the relatedness of these genome sequences using an alignment-free method based on the $D_{2}^{S}$ statistic ^29, 30^. We computed a $D_{2}^{S}$ distance, *d* for each possible pair of 143 genomes based on the presence of shared 25-mers using jD2Stat version 1.0 ( http://bioinformatics.org.au/tools/jD2Stat/) ^26^ and following Bernard *et al.* ^25^. Here the distance *d* is normalised based on genome sizes and the probabilities that corresponding *k*-mers occur in the compared sequences ^29, 30^; *d* ranges between 0.0 (i.e. two genomes are identical) and 15.5 (< 0.0001% 25-mers are shared between the two genomes). For a pair of genomes *a* and *b*, we transformed *d _ab_* into a similarity measure *S _ab_*, in which *S _ab_* = 10 – *d _ab_*. We ignore instances of *d* >10, as these pairs of sequences share ≤ 0.01% of 25-mers (i.e. there is little evidence of homology). To visualise the phylogenetic relatedness of these genomes, we adopted the D3 JavaScript library for data-driven documents ( https://d3js.org/). In this network, each node represents a genome, and an edge connecting two nodes represents the qualitative evidence of shared *k*-mers between them. We set a threshold function *t* for which only edges with *S* ≥ *t* are displayed on the screen. Changing *t* dynamically changes the network structure. The resulting dynamic network is available at http://bioinformatics.org.au/tools/AFnetwork/.

## Results and discussion

**Figure 1.**
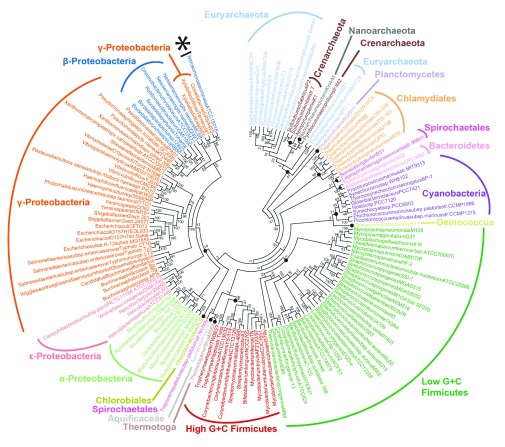
The alignment-free phylogenetic tree topology of the 143 Bacteria and Archaea genomes based on $D_{2}^{S}$ statistic, modified based on the tree in Bernard *et al.* ^25^; jackknife support at each internal node is shown. Each phylum is represented in a distinct colour, and the backbones identified in Beiko *et al.* ^10^ are shown on the internal node with black filled circles. The association of *Coxiella burnetii* and *Nitrosomonas europaea* is marked with an asterisk.

Figure 1 shows the phylogenetic tree of the 143 Bacteria and Archaea genomes that we previously inferred using an alignment-free method based on the $D_{2}^{S}$ statistic ^29, 30^. In an earlier study ^10^, a supertree was generated for these genomes, summarising 22,432 protein phylogenies. Incongruence between the two trees was observed in 42% of the bipartitions, most of which are at terminal branches ^25^. The alignment-free tree ( Figure 1) recovers 13 out of the 15 “backbone” nodes ^10^, distinct clades of Archaea and Bacteria, a monophyletic clade of Proteobacteria, and the lack of resolution between gamma- and beta-Proteobacteria, in agreement with previously published studies; as such, this tree captures most of the major biological groupings of Bacteria and Archaea as presently understood.

**Figure 2.**
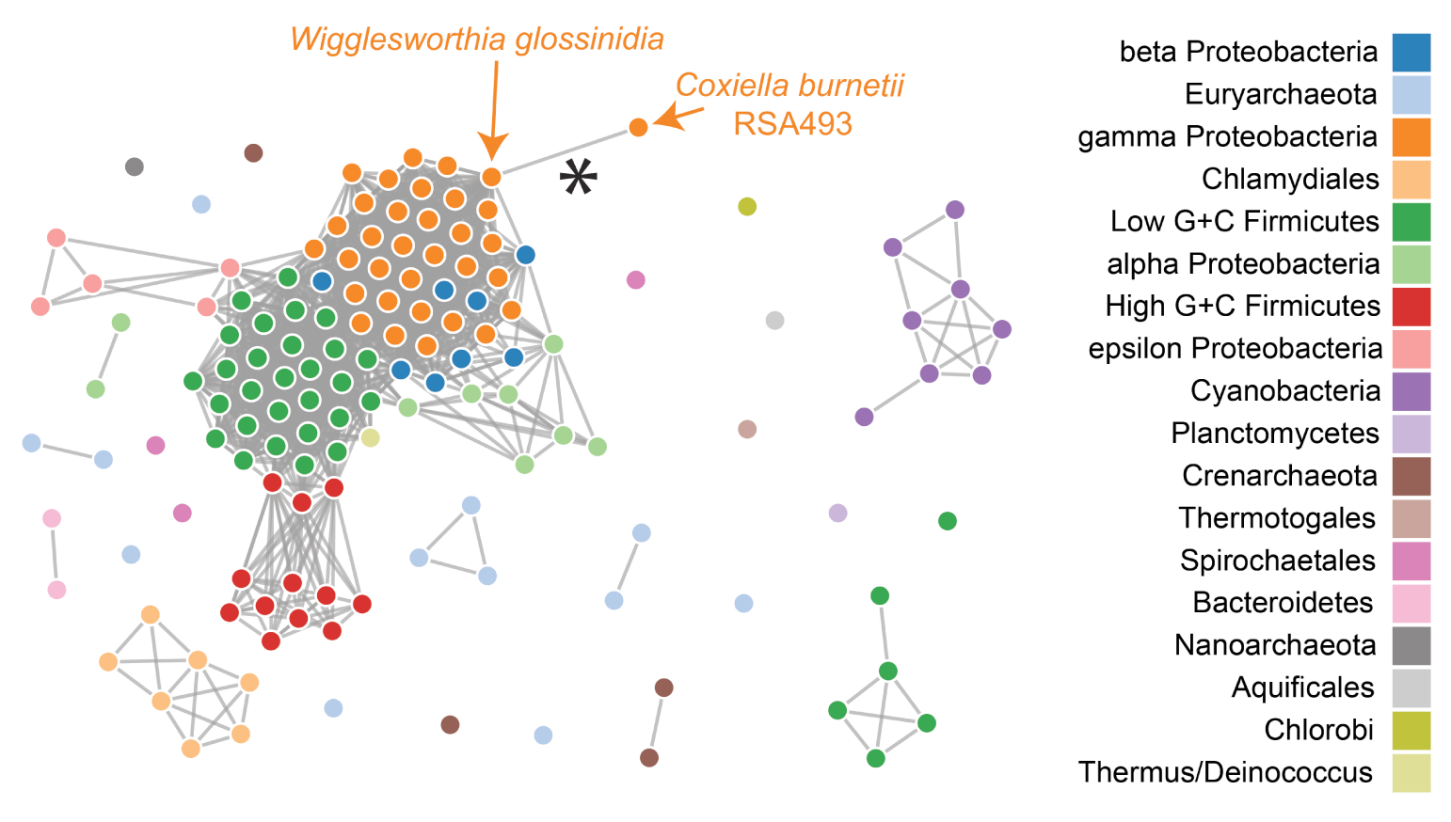
Alignment-free phylogenetic network of the 143 Bacteria and Archaea genomes based on $D_{2}^{S}$ statistic using 25-mers, at *t* = 2. Each phylum is represented in a distinct colour, each node represents a genome and an edge represents a qualitative evidence of shared 25-mers between two genomes. The association between *Coxiella burnetii* and *Wigglesworthia glossinidia* is marked with an asterisk.

Figure 2 shows the network of phylogenetic relatedness of the same 143 genomes; a dynamic view of this network is available at http://bioinformatics.org.au/tools/AFnetwork/. As in our tree ( Figure 1), Archaea and Bacteria form two separate paracliques; even at *t* = 0, we found only one archaean isolate (the euryarchaeote *Methanocaldococcus jannaschii* DSM 2661) linked to the bacterial groups Thermotogales and Aquificales ^25^. Upon reaching *t* = 3, most of the 14 phyla have formed distinct densely connected subgraphs in our network, i.e. Cyanobacteria and Chlamydiales form cliques at *t* = 1.5 and all subgroups of Proteobacteria form a large paraclique with the Firmicutes at *t* = 2. Four *Escherichia coli* and two *Shigella* isolates, known to be closely related, form a clique up to *t* = 8.5. Interestingly, this network also showcases the extent that genomic regions are shared among diverse phyla, e.g. the high extent of genetic similarity among Proteobacteria *versus* the low extent between Chlamydiales and Cyanobacteria. Our observations largely agree with published studies ^10, 25^, but also highlight the inadequacy of representing microbial phylogeny as a tree. For instance, in the tree *Coxiella burnetii*, a member of the gamma-Proteobacteria, is grouped with *Nitrosomonas europaea* of the alpha-Proteobacteria (marked with an asterisk in Figure 1); in the network, the strongest connection of *C. burnetii* is with *Wigglesworthia glossinidia*, a member of the gamma-Proteobacteria (marked with an asterisk in Figure 2) at *t* = 2. Both *W. glossinidia* and *C. burnetii* are parasites; the *W. glossinidia* genome (0.7 Mbp) is highly reduced ^31^ and the *C. burnetii* genome (2 Mbp) is proposed to be undergoing reduction ^32^. As both the tree ( Figure 1) and network presented here were generated using the same alignment-free method, the contradictory position of *C. burnetii* is likely caused by the neighbour-joining algorithm used for tree inference ^25^. In this scenario, the *C. burnetii* genome connects with *N. europaea* because it shares high similarity with *N. europaea* and *Neisseria* genomes of the beta-Proteobacteria ( *S* between 1.43 and 1.68), second only to *W. glossinidia* ( *S* = 2.05), and because it shares little or no similarity with other genomes of gamma-Proteobacteria that are closely related to *W. glossinidia*, i.e. *Buchnera aphidicola* isolates (average *S* = 0.63) and “ *Candidatus* Blochmannia floridanus” ** ( *S* = 0).

By changing the threshold *t*, we can dynamically visualise changes in the network structure. These changes are not random, but appear to correlate to the evolutionary history of the species. At *t* = 0, Archaea and Bacteria form two distinct paracliques, linked only by two edges, and the Planctomycetes isolate forms a singleton. When we increase *t* from 1 to 2, the Archaea and Bacteria paracliques quickly dissociate from each other; within the Bacteria, cliques of Chlamydiales and Cyanobacteria are formed and the Spirochaetales become isolated. Going from *t* = 2 to *t* = 3 we observe a scission between Firmicutes and Proteobacteria, and at *t* > 3 all classes of Proteobacteria start to form respective paracliques. The separation (as *t* is incremented) of a densely connected subgraph involving all representatives of a phylum, from the rest of the network mimics the divergence of this phylum from a common ancestor. Because the similarity measures do not have a unit (such as number of substitutions per site), it is not straightforward to interpret *S* as an evolutionary rate or divergence time. A comprehensive comparative analysis between our network here and one that is generated using multiple sequence alignment is beyond the scope of this work. However, our findings suggest that our alignment-free network yields snapshots of biologically meaningful evolutionary relationship among these genomes, and that increasing the threshold based on the proportion of shared *k*-mers recapitulates the progressive separation of genomic lineages in evolution.

The alignment-free network reconstructed using whole-genome sequences thus recovers phylogenetic signals that cannot be captured in a binary tree. Using this approach, we generated the network in < 30 minutes; a whole-genome alignment of 143 sequences would have taken days, and even then, the alignment would be difficult to interpret given the genome dynamics in Bacteria and Archaea ^9– 13, 33^. One can imagine inferring a network of thousands of microbial genomes in a few hours using distributed computing. More importantly, the network can be visualised dynamically, explored interactively and shared.

Other biological questions could be addressed by linking the *k*-mers to their genomic locations and annotated genome features, e.g. in a relational database ^34^. For instance, we could use such a database to compare thousands of isolates and identify core gene functions for a specific phylum or genus, or exclusive *versus* non-exclusive functions in bacterial pathogens, in a matter of seconds. We can also use *k*-mers to quickly search for biological information e.g. functions relevant to lateral genetic transfer, recombination or duplications.

In contrast to Haeckel’s “Biogenetic Law”, *k*-mers used in this way recapitulate phylogenetic signal, not ontogeny. Alignment-free approaches generate a biologically meaningful phylogenetic inference, and are highly scalable. More importantly, representing alignment-free phylogenetic relationships using a network captures aspects of evolutionary histories that are not possible in a tree. As more genome data become available, Haeckel’s goal of depicting the History of Life is closer to reality.

## Data availability

The data referenced by this article are under copyright with the following copyright statement: Copyright: © 2016 Bernard G et al.

The 143 Bacteria and Archaea genomes used in this work are the same dataset used in an earlier study ^25^, available at http://dx.doi.org/10.14264/uql.2016.908 ^35^. The dynamic phylogenetic network of these genomes is available at http://bioinformatics.org.au/tools/AFnetwork, with the source code available at http://dx.doi.org/10.14264/uql.2016.952 ^36^
