# Supplementary material for: Recapitulating phylogenies using k-mers: from trees to networks
Source: F1000Res. 2016 Dec 23;5:2789. Originally published 2016 Nov 29. [Version 2] doi: 10.12688/f1000research.10225.2 (PMC5224691; doi:10.12688/f1000research.10225.2)
Supplement: Peer review history

# Peer review: Referee response for version 2

**DOI:** 10.5256/f1000research.11322.r18754

**Published:** 2016-12-23

**Authors:** Weilong Hao

**Affiliations:** 1 Department of Biological Sciences, Wayne State University, Detroit, MI, USA

The authors' responses are acceptable.

I have read this submission. I believe that I have an appropriate level of expertise to confirm that it is of an acceptable scientific standard.

# Peer review: Referee response for version 1

**DOI:** 10.5256/f1000research.11014.r18060

**Published:** 2016-12-13

**Authors:** Weilong Hao

**Affiliations:** 1 Department of Biological Sciences, Wayne State University, Detroit, MI, USA

The manuscript uses *k*-mers from whole-genome sequences to recapitulate phylogenetic relationships from trees to networks. The analyses seemed to be convincing, and of general interest. I just have some comments on the manuscript structure and some other minor suggestions.

 The authors used Ernst Haeckel’s phylogeny and Biogenetic Law to start their manuscript. Although it is fun to read all these historical pieces, the link between Haeckel’s ideas and the construction of networks using *k*-mers was not made strong in the current version of the manuscript.

 The authors compared alignment-free data against sequence alignments, and stated that the sequence alignment approach “ignores important evolutionary processes that are known to shape the genomes of microbes” followed by mentioning recombination, genome rearrangement, and lateral gene transfer. This is not accurate, as sequence alignments can also be used to reconstruct web-like phylogenetic relationships, which are sometimes called phylogenetic networks (e.g., Huson and Bryant 2006). I think it is important to carefully define and compare the networks mentioned in this manuscript and the phylogenetic networks mentioned by Huson and Bryant. Along this line, approaches based on sequence alignments might not all assume tree-like relationship. Furthermore, the authors mentioned evolutionary events, such as recombination, genome rearrangement, and lateral gene transfer, that are difficult to study using sequence alignments, but did not provide detailed evidence on whether *k*-mers can tackle them all. I suggest the authors to rather stay closer to their data and make more specific statements.

 In the third introduction paragraph, “By default, it is assumed that the best alignment can be achieved simply by displaying the sequences in the same direction and inserting gaps where needed. This assumption is largely valid when working with exons or proteins of morphologically complex eukaryotes. However, in microbes this assumption is violated...” I feel the meaning of “assumption” in each of these sentences is a moving target. If they are talking about orthologous sequences, the analysis of orthologs should hold for both eukaryotes and prokaryotes. The key here, I guess, is the comparison of ortholgs, versus, the comparison of exenologs even non-homologs. Another minor point is the use of “microbes”, which can mean, bacteria, archaea, and small-eukaryotes. I don’t think it is a good word to use here.

 The authors did not justify the use of the 143 genomes. It seemed that they were inherited from their previous study conducted some time ago, and likely skewed in terms of taxon-sampling. Since taxon-samping is important for tree-like phylogenetic analysis, it would be nice to address how the improved (or more balanced) taxon-sampling can benefit the network analyses.

 The authors wrote “... in agreement with previously published studies; as such, this tree represents reality as presently understood, i.e., is biologically correct”. The use of words such as reality, biologically correct here, is inappropriate.

 The data of Wigglesworthia, Coxiella and others are of potential interest. The readers would definitely appreciate some real data analyses to address them, which are currently lacking.

 The cited references are relatively recent and skewed. Some of the older and more influential papers need to be added (for both networks and alignment free).

I have read this submission. I believe that I have an appropriate level of expertise to confirm that it is of an acceptable scientific standard, however I have significant reservations, as outlined above.

# Response

**Published:** 2016-12-15

**Authors:** Cheong Xin Chan

**Affiliations:** Institute for Molecular Bioscience, The University of Queensland, Australia

Thank you for these comments.

- **The link between Haeckel’s ideas and the construction of networks using *k*-mers was not made strong in the current version of the manuscript.**

The work we present here is a proof-of-concept for a biologically informative network based on *k*-mers extracted from whole-genome sequences. We hope to convince readers that dynamic visualization of such a network is intuitive for exploring and addressing biological questions, aiding discovery. The paper is part of a special collection of F1000Research articles in phylogenetics, commemorating the 150th anniversary of Ernst Haeckel’s Tree of Life published in 1866. Here we argue that by using *k*-mers we can recapitulate phylogenetic signal, somewhat in the same spirit as Haeckel famously argued that “ontogeny recapitulates phylogeny”. More precisely, our claim is that “increasing the threshold based on the proportion of shared *k*-mers recapitulates the progressive separation of genomic lineages in evolution”. Full consideration of Haeckel’s work in the context of Darwinian evolution then and today is well beyond the scope of our brief paper, although we cite some key references.

- **The authors … stated that the sequence alignment approach “ignores important evolutionary processes that are known to shape the genomes of microbes” followed by mentioning recombination, genome rearrangement, and lateral gene transfer. This is not accurate, as sequence alignments can also be used to reconstruct web-like phylogenetic relationships, which are sometimes called phylogenetic networks (e.g., Huson and Bryant 2006). I think it is important to carefully define and compare the networks mentioned in this manuscript and the phylogenetic networks mentioned by Huson and Bryant.** **Along this line, approaches based on sequence alignments might not all assume tree-like relationship.**

We agree and have now rewritten part of the Abstract to stage our argument more clearly: genomic processes in microbes can undermine the assumptions that underlie multiple sequence alignment, hence phylogenetic inference as usually practiced. We have now cited other articles on phylogenetic networks in the text where appropriate, specifically Huson and Bryant ^1^ and Kunin *et al.* ^2^. Comprehensive comparison of *k*-mer-based and (alignment-based) phylogenetic networks is important but, due to its complexity, beyond the scope of this paper; we have now clarified this in the revised text.

- **The authors mentioned evolutionary events, such as recombination, genome rearrangement, and lateral gene transfer … but did not provide detailed evidence on whether k-mers can tackle them all. I suggest the authors to rather stay closer to their data and make more specific statements.**

In Chan *et al.* ^3^ and Bernard *et al.* ^4^ we provided detailed evidence that alignment-free approaches based on *k*-mers, at multi-genome scale, can be robust to insertions/deletions, genome rearrangement and lateral genetic transfer; these articles are cited where appropriate.

- **In the third introduction paragraph, “By default, it is assumed that the best alignment can be achieved simply by displaying the sequences in the same direction and inserting gaps where needed. This assumption is largely valid when working with exons or proteins of morphologically complex eukaryotes. However, in microbes this assumption is violated...” I feel the meaning of “assumption” in each of these sentences is a moving target. If they are talking about orthologous sequences, the analysis of orthologs should hold for both eukaryotes and prokaryotes. **

We have now revised the text to make it clear that the main assumption underlying multiple sequence alignment, i.e. that the alignment columns display homology position-by-position along the length of the sequences, is largely valid when working with highly conserved orthologs of any source; and that the validity of this assumption is often undermined in the case of microbial genome sequences, due to recombination and rearrangement.

- **Another minor point is the use of “microbes”, which can mean, bacteria, archaea, and small-eukaryotes. I don’t think it is a good word to use here.**

We used the word “microbes” here specifically to include archaea, bacteria and microbial eukaryotes. Genomes of many microbial eukaryotes are known to be impacted by lateral genetic transfer, at frequencies sometimes nearly as large as in bacteria and archaea.

- **The authors did not justify the use of the 143 genomes. … Since taxon-sampling is important for tree-like phylogenetic analysis, it would be nice to address how the improved (or more balanced) taxon-sampling can benefit the network analyses.**

Here we used the 143-genome dataset because the phylogenetic relationships among these genomes have been studied using careful alignment-based methods ^5^ and by alignment-free approaches ^4^; it thus provides a good reference for comparison. We have now clarified this in the text. In our alignment-free network, each edge represents the qualitative evidence of *k*-mers shared pairwise between two genomes. This evidence is not affected by other genomes present in (or absent from) the dataset. Therefore, our networks are not affected by taxon-sampling biases of the sort encountered in tree inference. Of course, the presence or absence of a critical node (genome) might affect the biological conclusion we draw from a network, but the same is true for any scientific analysis. We considered the effect of phyletic balance on the inference of lateral genetic transfer networks in another context ^6^.  

- **The authors wrote “... in agreement with previously published studies; as such, this tree represents reality as presently understood, i.e., is biologically correct”. The use of words such as reality, biologically correct here, is inappropriate.**

We agree and now state that “as such, this tree captures most of the major biological groupings of Bacteria and Archaea as presently understood”.

- **The data of *Wigglesworthia*, *Coxiella* and others are of potential interest. The readers would definitely appreciate some real data analyses to address them, which are currently lacking.**

A follow-up analysis between *Wigglesworthia* and *Coxiella* would indeed be interesting, but is beyond the scope of this Research Note, the aim of which is to present limited findings in hopes of inspiring and encouraging others to explore this research area.

- **Some of the older and more influential papers need to be added (for both networks and alignment free).**

We have now cited older, relevant references in the text for both networks ^1, 2^ and alignment-free methods ^7^.

**References**

1. Huson DH, Bryant D: Application of phylogenetic networks in evolutionary studies. *Mol Biol Evol*. 2006; **23**(2): 254-67.
2. Kunin V, Goldovsky L, Darzentas N *, et al.*: The net of life: reconstructing the microbial phylogenetic network. *Genome Res*. 2005; **15**(7): 954-9.
3. Chan CX, Bernard G, Poirion O *, et al.*: Inferring phylogenies of evolving sequences without multiple sequence alignment. *Sci Rep*. 2014; **4**: 6504.
4. Bernard G, Chan CX, Ragan MA: Alignment-free microbial phylogenomics under scenarios of sequence divergence, genome rearrangement and lateral genetic transfer. *Sci Rep*. 2016; **6**: 28970.
5. Beiko RG, Harlow TJ, Ragan MA: Highways of gene sharing in prokaryotes. *Proc Natl Acad Sci U S A*. 2005; **102**(40): 14332-7.
6. Cong Y, Chan YB, Ragan MA: Exploring lateral genetic transfer among microbial genomes using TF-IDF. *Sci Rep*. 2016; **6**: 29319.
7. Domazet-Lošo M, Haubold B: Alignment-free detection of local similarity among viral and bacterial genomes. *Bioinformatics*. 2011; **27**(11): 1466-72.

# Peer review: Referee response for version 1

**DOI:** 10.5256/f1000research.11014.r18402

**Published:** 2016-12-12

**Authors:** Bernhard Haubold

**Affiliations:** 1 Department Evolutionary Genetics, Max Planck Institute for Evolutionary Biology, Plön, Germany

Phylogeny reconstruction is a classical research topic in bioinformatics. In this context the standard trade-off between speed and accuracy becomes a choice between slow but accurate sequence alignment on the one hand and fast but less accurate alignment-free methods on the other. Bernard *et al.* aim for speed and use an established alignment-free measure, D_2, to reconstruct the phylogeny of 143 Bacteria and Archaea from full genome sequences. D_2 is based on the number of shared *k*-mers, and the main contribution of the paper is the visualization of the D_2 distance matrix of the 143 taxa as a network rather than the traditional bifurcating tree. This visualization is dynamic in the sense that the user can choose a similarity threshold between 0 and 10, and watch as the taxa disintegrate from initially two clusters to essentially every taxon on its own. This is an innovative way of presenting large-scale evolutionary relationships, and the tool is fun to use. As the authors remark, it is unclear how the D_2 metric scales with more familiar measures of evolutionary time such as substitutions per site. It would thus be interesting to explored this in future work; for example by supplying a version of the visualization tool that allows users to upload their own sequences. I was also wondering how the networks generated by Bernard *et al.* compare to established methods of network-based evolutionary analysis such as SplitsTree and minimum spanning trees. I realize that these are both usually based on alignments, but it is always possible to analyze a given alignment using D_2, thereby allowing a direct assessment of the accuracy lost (if any) for the speed gained.

I have read this submission. I believe that I have an appropriate level of expertise to confirm that it is of an acceptable scientific standard.

# Response

**Published:** 2016-12-15

**Authors:** Cheong Xin Chan

**Affiliations:** Institute for Molecular Bioscience, The University of Queensland, Australia

Thank you for these comments. Indeed, the correlation between D2 metrics and evolutionary distances is an interesting area, and a tool that allows users to upload their own datasets would be useful. A comparative analysis between a *k*-mer-based network and a phylogenetic network based on multiple sequence alignment, although doable, is not straightforward. We believe the adoption of alignment-free methods in phylogenetic inference is still in its infancy, and we hope that this work will inspire and encourage other researchers to pursue this approach.
